# TMEM132A: a novel susceptibility gene for lung adenocarcinoma combined with venous thromboembolism identified through comprehensive bioinformatic analysis

**DOI:** 10.3389/fonc.2025.1564114

**Published:** 2025-05-13

**Authors:** Pei Xie, Yingli Liu, Pingping Bai, Yue Ming, Qi Zheng, Li Zhu, Yong Qi

**Affiliations:** Department of Pulmonary and Critical Care Medicine, Zhengzhou University People’s Hospital, Henan Provincial People’s Hospital CN, Zhengzhou, China

**Keywords:** lung adenocarcinoma, venous thromboembolism, Lasso algorithm, immune infiltration, diagnostic biomarker

## Abstract

**Background:**

Mounting evidence indicates that lung adenocarcinoma (LUAD) patients are at elevated risk for venous thromboembolism (VTE), presenting a major clinical challenge. This study utilized public databases to identify crosstalk genes (CGs) between LUAD and VTE, applied machine learning methods to discover shared diagnostic biomarkers, and explored their underlying mechanisms.

**Methods:**

Disease-specific genes for VTE were extracted from comprehensive genomic databases (CTD, DisGeNET, GeneCards, OMIM), while transcriptomic profiles of LUAD and VTE cohorts were retrieved from GEO via GEOquery implementation. Molecular crosstalk analysis identified candidate genes through differential expression algorithms and disease-association metrics. Functional annotation employed GO and KEGG analyses to elucidate the biological significance of identified CGs. LASSO regression analysis of VTE and LUAD matrices yielded overlapping diagnostic biomarkers. Immune contexture was characterized via CIBERSORT deconvolution, followed by correlation analyses between hub genes and immune infiltration profiles. Hub genes expression was corroborated through independent cohort validation and serological quantification. Diagnostic utility was evaluated through receiver operating characteristic (ROC) curve and nomogram. Therapeutic potential was assessed via DSigDB-based drug sensitivity profiling.

**Result:**

Through transcriptomic analysis, we identified 381 CGs, which demonstrated significant enrichment in inflammatory cascades, immunological processes, and coagulation pathways. LASSO regression analysis of LUAD and VTE cohorts revealed TIMP1 and TMEM132A as putative shared diagnostic biomarkers. TMEM132A exhibited significant correlation with immune cell infiltration patterns across both diseases, modulating the immune microenvironment. Validation cohorts and serological assessment confirmed elevated TMEM132A expression in LUAD and LUAD combined with VTE phenotypes. The diagnostic accuracy of TMEM132A was substantiated by ROC curves and nomogram analyses. Pharmacological sensitivity analysis indicated that TMEM132A may serve as a potential target for the therapeutic agents birabresib and abemaciclib.

**Conclusion:**

TMEM132A demonstrates diagnostic utility as a predictive biomarker for VTE occurrence in LUAD, suggesting its potential role as a susceptibility gene in this patient cohort.

## Introduction

Venous thromboembolism (VTE), including deep vein thrombosis (DVT) and pulmonary embolism (PE), is a multifactorial disease which ranked the third most common vascular disease after acute myocardial infarction and stroke and affects 10 million people each year (1–4). Lung cancer is one of the most prevalent and deadliest malignancies worldwide, with a 3-year survival rate have increased from 26% to 40% because of multi-therapies like immunotherapy, chemotherapy (5). The main pathological types of lung cancer are non-small cell lung cancer (NSCLC) and small cell lung cancer (SCLC), with LUAD accounting for nearly 40% of NSCLC cases (6, 7). Studies indicate that VTE is a common clinical complication associated with NSCLC (8–10). Research indicates a strong association between VTE and histological subtypes of NSCLC, with LUAD identified as a high-risk factor for VTE, showing a significantly higher incidence than that observed in lung squamous cell carcinoma (8, 11–13). Dickmann et al. demonstrated advanced stage, regional, and distant lymph node metastasis in patients with cancer significantly increased the risk of VTE (14). VTE portends a poor prognosis for patients with lung cancer and increases mortality and medical financial burden (15).

Mechanistically, the hypercoagulable state of blood caused by malignant tumors and endothelial cell activation can cause the occurrence of venous thrombosis (16). The hypercoagulable state of malignant tumors may be driven by pathways unique to cancer. In particular, the presence of malignant tumors often increases the activation of the coagulation cascade and platelets, as well as the circulating levels of certain blood cells (such as platelets and leukocytes) (17). In addition, cancer treatment (including chemotherapy and new targeted therapies) may increase thrombosis through mechanisms that are not yet fully understood (18). Finally, patient characteristics (such as age, gender, and history of thrombosis) and comorbidities (such as sepsis) are also determinants of the hypercoagulable state of blood in cancer patients (17). Based on this, timely risk assessment and screening can be performed for cancer patients with corresponding risks in the clinic, so that the formation of venous thrombosis can be prevented in time, ultimately improving the quality of life of cancer patients.

**Figure 1:**
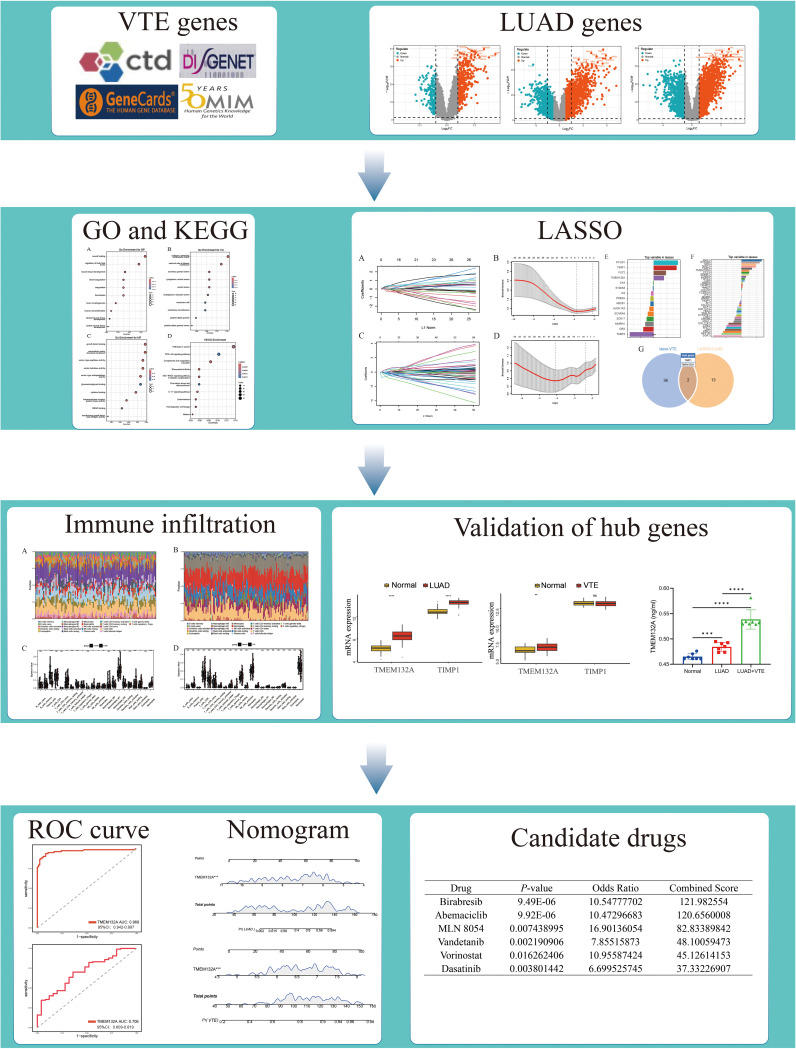
Workflow chart.

A large number of studies have shown a close association between LUAD and VTE. However, the underlying mechanisms remain multifactorial and needs deeper research. Herein, this research employs bioinformatics analyses on LUAD and VTE cohorts to elucidate their relationship and investigate the potential biological processes, while also providing a theoretical basis for the susceptibility of LUAD patients to VTE. The study procedure is shown in **Figure 1**.

## Materials and methods

### Data acquisition and processing

VTE-related genes were sourced from four databases: the CTD (http://ctdbase.org/), DisGeNET (https://www.disgenet.org/), GeneCards (https://www.genecards.org/), and OMIM (http://omim.org/) databases. The data collection was completed by July 7, 2024. Gene expression data were obtained from the GEO database (https://www.ncbi.nlm.nih.gov/geo/). Five LUAD datasets were analyzed: GSE10072, GSE32863, GSE40791, GSE43458 and GSE46539. To estimate the diagnostic efficiency, we also downloaded GSE75037 and GSE48000. All raw CEL files underwent log2 transformation for normalization.

### Clinical sample collection

Clinical samples were verified subsequently. Blood samples were collected from three distinct groups: 7 patients with advanced LUAD, 7 patients with advanced LUAD combined with VTE, and 7 healthy subjects. Samples were drawn from whole blood, with LUAD diagnosis confirmed through pathological biopsy, blood examinations, and imaging. VTE was identified via blood examinations and imaging. Advanced LUAD patients and healthy controls were age and sex-matched with the patients having advanced LUAD combined with VTE. The blood samples collection place at Henan Provincial People’s Hospital (Henan, China). The blood was allowed to clot at room temperature for 30 minutes before being centrifuged at 2500 × g for 10 minutes to separate the serum. The resulting serum was aliquoted and stored at -80°C until analysis. The study was approved by the Ethics Committee of Henan Provincial People’s Hospital (Ethical Review 2023 (97)).

### Identification of CGs

The top 700 VTE-associated genes were curated from the CTD, DisGeNET, GeneCards, and OMIM databases according to their respective scoring criteria. Where fewer than 700 genes were listed in a database, all entries were included. These VTE-related genes obtained from disease-related databases were merged and de-duplicated to obtain the VTE genes. Download the GEO dataset and use the corresponding probes to annotate and remove duplicates. Then perform log2 normalization on the GEO dataset. The “limma” R package was used to screen the differentially expressed genes (DEGs) from the GSE10072, GSE32863, GSE40791, GSE43458 and GSE46539 datasets (19). The selection criteria of DEGs in the five datasets were set as |log FC| ≥ 1 and *p*-value < 0.05&FDR <0.05. Subsequently, the DEGs filtered based on these criteria were consolidated and de-duplicated to obtain the LUAD genes. Subsequently, the VTE genes and LUAD genes were intersected to identify the CGs of the two diseases for further analysis.

### Functional enrichment analysis

Gene Ontology (GO) and Kyoto Encyclopedia of Genes and Genomes (KEGG) pathway enrichment analyses were conducted by the “clusterProfile” R package (20, 21). The significant differential GO and KEGG terms were defined with a strict cut-off of *p* < 0.05.

### Machine learning-based hub gene screening

LASSO analysis was were employed to screen for hub genes in LUAD and VTE. LASSO analysis was performed using the “glmnet” package of R software and the optimal value of the penalty parameter was determined by 10-fold cross-validation (22, 23). Ultimately, the intersection CGs was selected as the hub genes to diagnose LUAD with VTE.

### Immune infiltration analysis

The distribution of immune cells between diseases and normal groups was explored using the CIBERSORT algorithm, which is a tool to calculate the relative percentage of immune cells based on gene expression matrix (24). Stacked column graphs are employed to visually depict the proportions of 22 immune cell types in LUAD and VTE. Additionally, column scatter plots are utilized to compare the levels of immune cell infiltration between LUAD and control groups, as well as between VTE and control groups. Meanwhile, correlation scatter plots provide an intuitive representation of the relationships between hub genes and the extent of infiltration of various immune cell types (25).

### Validation of hub genes

The GSE75037 and the GSE48000 dataset were used for the validation of the candidate hub genes in LUAD and VTE. GEOquery package were used to obtain the expression data of hub genes (19).

ELISA sandwich method using human ELISA assay kit (MLBIO, Shanghai, China) was used to determine the concentration of TMEM132A in serum in different groups of patients, and all steps were carried out strictly according to the manufacturer’s instructions. The study was approved by the Ethics Committee of Henan Provincial People’s Hospital (Ethical Review 2023 (97)).

### ROC evaluation and nomogram construction

The “pROC” R package was performed to ROC analysis, for evaluating the diagnostic performance of hub genes by calculating the area under the curve (AUC). AUC > 0.5 was considered to have diagnostic value (26). A nomogram was constructed using the “rms” R package to ascertain the significance of hub genes in diagnosing LUAD and VTE (25). The nomogram comprises “Points” and “Total Points”, with “Points” representing the individual scores assigned to candidate genes and “Total Points” denoting the cumulative scores of all candidate genes.

### Identification of drug candidates

The common core hub genes of LUAD and VTE were uploaded to the Enrichr platform (https://maayanlab.cloud/Enrichr/) (27), following which the Drug Signature Database (DSigDB) was utilized to identify candidate drugs associated with core hub gene (28).

### Statistical analysis

GraphPad Prism (version 9.0) and R software (version 4.4.1) were used for data processing, analysis, and visualization. Univariate variables were analyzed by Wilcoxon test. Correlation analysis was conducted using the Spearman test. ROC curve analysis was performed using the “pROC” R package, and the diagnostic performance was evaluated by calculating the area under the curve (AUC). The “rms” R package was used to draw nomogram. *p*-value < 0.05 was considered statistically significant.

## Results

### Identification of CGs in LUAD and VTE cohorts

**Figure 2:**
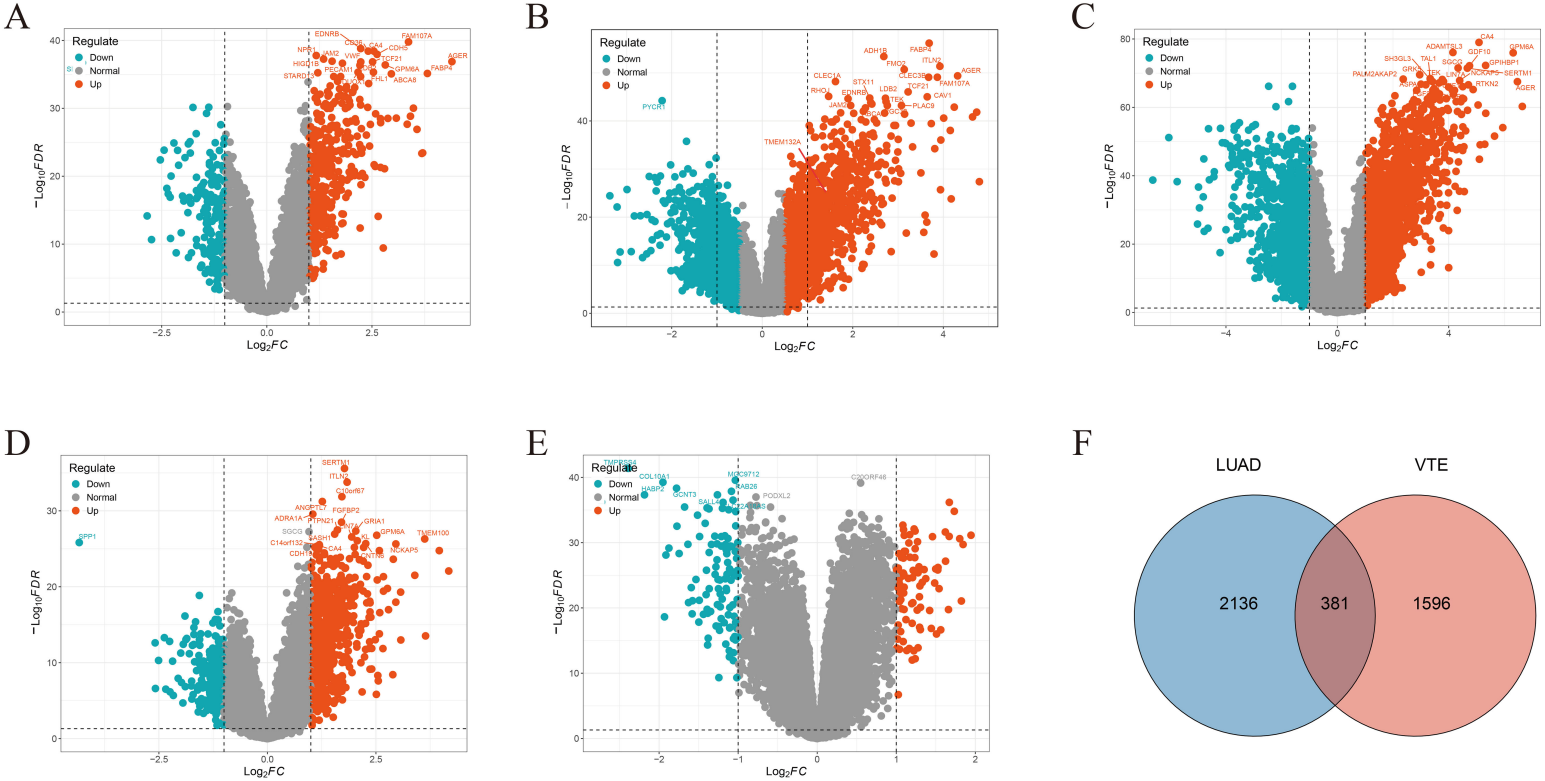
Crosstalk genes (CGs) in LUAD and VTE. Volcano plots showed DEGs in **(A)** GSE10072, **(B)** GSE32863, **(C)** GSE40791, **(D)** GSE43458, **(E)** GSE46539. **(F)** Venn plots of the CGs between LUAD genes with VTE genes.

We acquired 700, 378, 700, and 624 VTE-related genes from the CTD, DisGeNET, GeneCards, and OMIM databases, respectively. Then, 1977 VTE genes were obtained by merging and deduplicating. In the LUAD datasets GSE10072, GSE32863, GSE40791, GSE43458 and GSE46539, a total of 450, 898, 1971, 823, and 175 DEGs, were identified respectively (**Figures 2A–E**). After merging and removing duplicates, 2517 LUAD genes were obtained from the DEGs identified in these five datasets. As the Venn diagram showed in **Figure 2F**, there were 381 overlapping CGs between LUAD and VTE cohorts. The specific screening process is shown in **Table 1**.

**Table 1:** Identification of VTE and LUAD genes.

Disease	Data sources	Amount of raw data	Filter condition	Amount of data after filtering and de-duplication	Merge and deduplicate	Crosstalk genes
VTE	CTD DisGeNET GeneCards OMIM	16059 378 2543 624	If the raw data are more than 700, then top 700 are taken; if the raw data are less than 700, then all are included.	700 378 700 624	1977	381
LUAD	GSE10072 GSE32863 GSE40791 GSE43458 GSE46539	12261 18127 17270 17962 17167	FDR<0.05 and |log2FoldChange|>1	450 898 1971 823 175	2517	

VTE, venous thromboembolism; LUAD, lung adenocarcinoma; FDR: false discovery rate.

### Enrichment analysis of CGs

**Figure 3:**
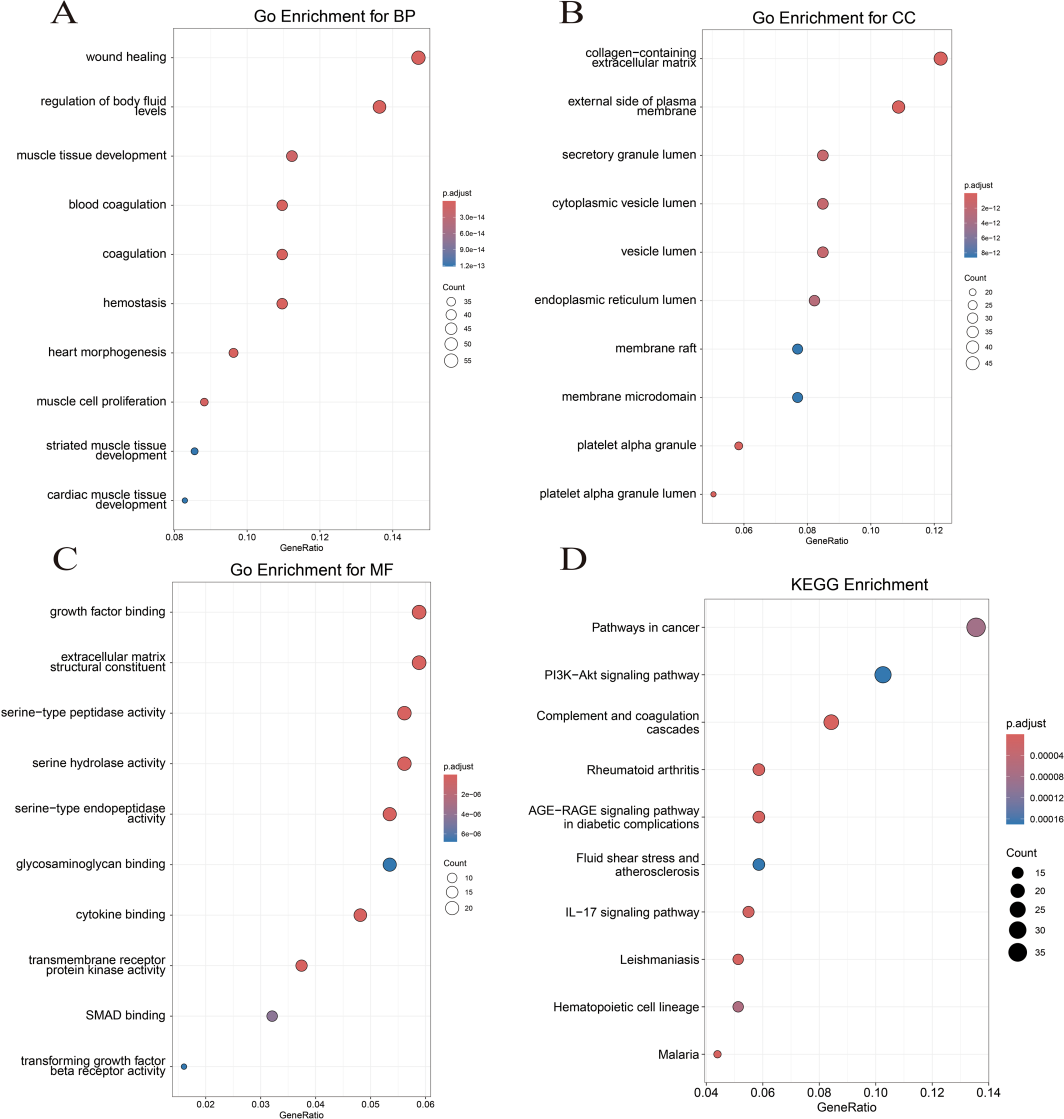
Function enrichment analysis. **(A)** Biological process of GO. **(B)** Cellular component of GO. **(C)** Molecular function of GO. **(D)** KEGG enrichment analysis of CGs.

GO and KEGG enrichment analyses were conducted to investigate the biological function of CGs. For biological processes, CGs predominantly concentrated in the signaling pathways of coagulation, hemostasis, and muscle cell proliferation (**Figure 3A**). Regarding cellular components, CGs were primarily enriched in the collagen-containing extracellular matrix, the external side of the plasma membrane, and platelet alpha granule (**Figure 3B**). In terms of molecular function, CGs showed significant enrichment in growth factor binding, cytokine binding, and extracellular matrix structural constituent (**Figure 3C**). KEGG enrichment analysis showed that CGs were significantly enriched in the pathways in cancer, PI3K-Akt signaling pathway, complement and coagulation cascades, and IL-17 signaling pathway (**Figure 3D**). These findings highlight the critical role of inflammation, immune response, and coagulation pathways in LUAD and VTE.

### Identification of hub gene

**Figure 4:**
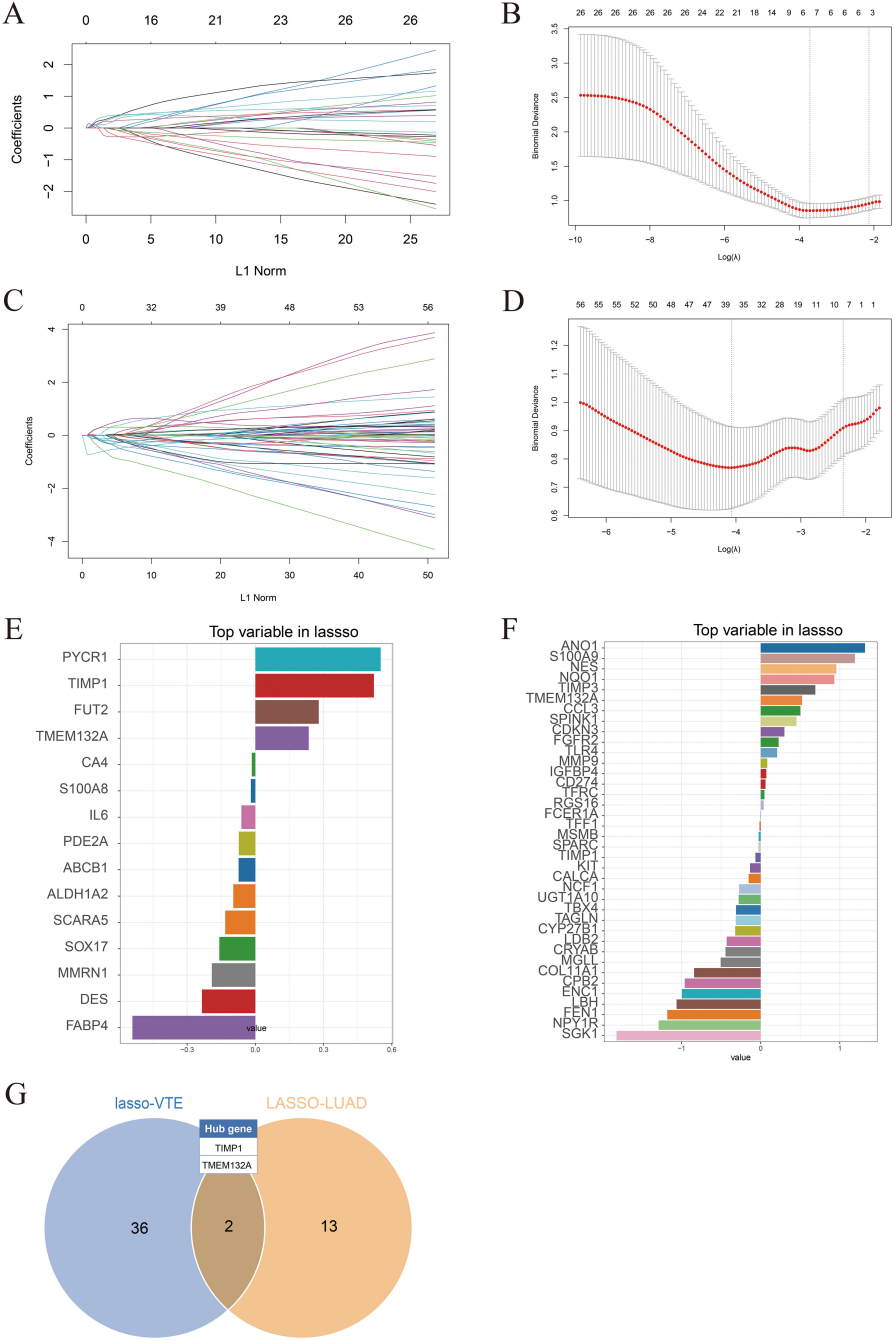
Identification of hub genes. **(A)** LASSO coefficient plots in the GSE75037 dataset. **(B)** 10-fold cross-validation plots in the GSE75037. **(C)** LASSO coefficient plots in the GSE48000 dataset. **(D)** 10-fold cross-validation plots in the GSE48000. **(E)** LASSO algorithm identified diagnostic characteristic genes related to LUAD. **(F)** LASSO algorithm identified diagnostic characteristic genes related to VTE. **(G)** Venn diagram showing hub genes identified in the LASSO model.

To identify potential candidate genes related to the diagnosis of LUAD and VTE, the LASSO algorithm was utilized to select characteristic genes. The non-zero coefficients in the LUAD dataset GSE75037 and the VTE dataset GSE48000 were selected by 10-fold cross-validation (**Figures 4A–D**). The LASSO algorithm identified 15 diagnostic CGs closely related to LUAD and 38 diagnostic CGs closely related to VTE (**Figures 4E, F**). Finally, two overlapping CGs (TIMP1 and TMEM132A) served as hub genes (**Figure 4G**).

### Immune cell infiltration and its correlation with hub genes

**Figure 5:**
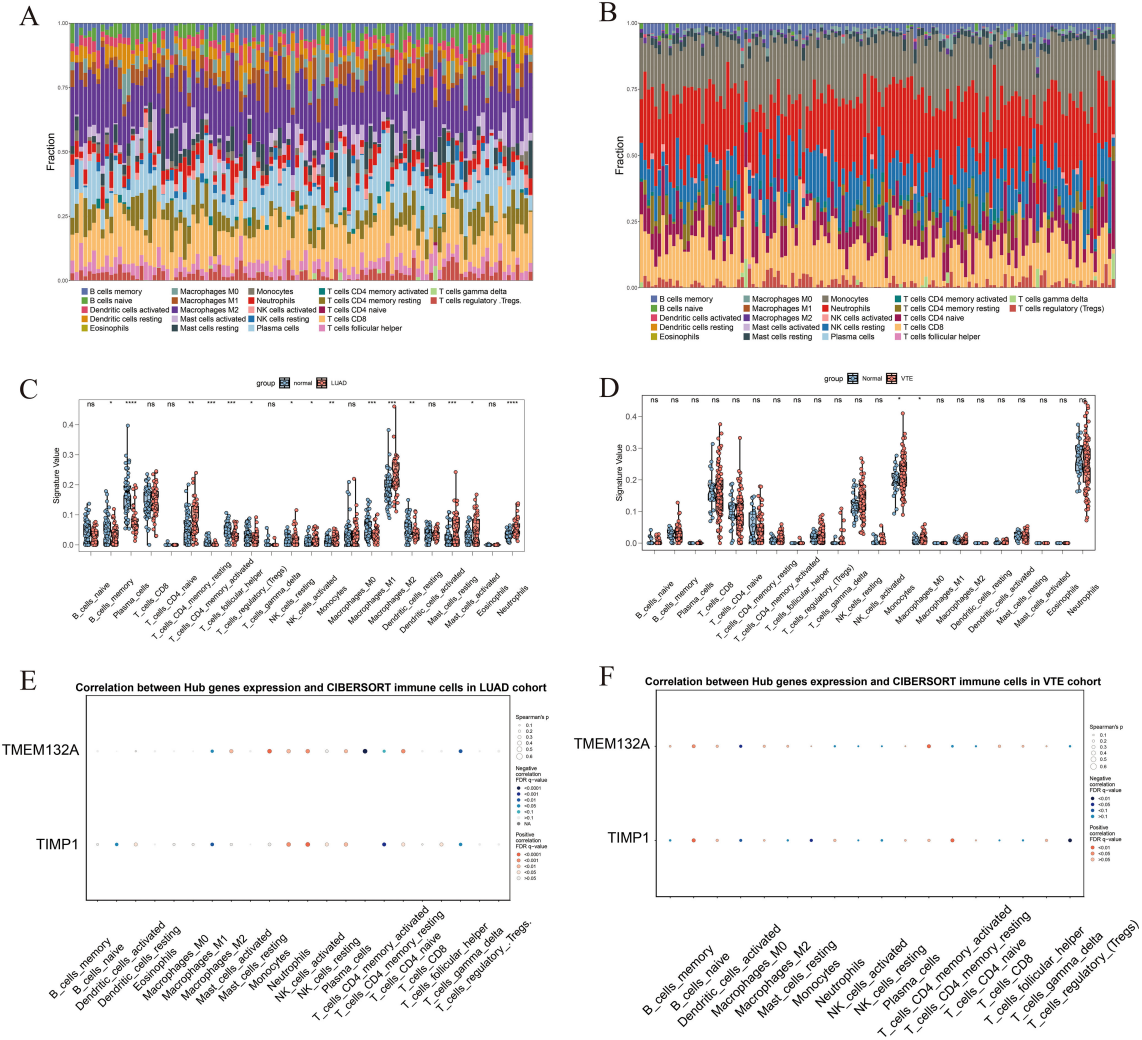
The immune infiltration landscape of LUAD and VTE. **(A)** The proportion of 22 immune cell types in LUAD samples visualized from the stacked column graph. **(B)** The proportion of 22 immune cell types in VTE samples visualized from the stacked column graph. **(C)** Immune cell infiltration differences between LUAD and control groups shown in the column scatter plot. **(D)** Immune cell infiltration differences between VTE and control groups shown in the column scatter plot. **(E)** Correlation between hub genes and immune cells infiltration levels shown in the correlation scatter plot in LUAD. **(F)** Correlation between hub genes and immune cells infiltration levels shown in the correlation scatter plot in VTE. *p < 0.05; **p < 0.01; ***p < 0.005; ****p < 0.001; ns, No statistical.

The CIBERSORT algorithm was used to analyze the differences in the immune microenvironment between patients and normal samples in the LUAD dataset GSE10072 and VTE dataset GSE48000. **Figures 5A** and **B** illustrate variations in the proportions of 22 immune cell types between LUAD and VTE. The stacked column graph in **Figure 5A** reveals elevated proportions of macrophages M2, plasma cells, and CD8 T cells in the LUAD group. **Figure 5B** shows higher proportions of monocytes, neutrophils, resting NK cells, and CD8 T cells in the VTE group. The column scatter plot presented the levels of resting CD4 memory T cells, resting NK cells, activated NK cells, monocytes, macrophages M2, resting mast cells, activated mast cells and neutrophils were higher in LUAD samples (**Figure 5C**). In VTE samples, higher levels of monocytes and macrophages M0 were observed compared with normal samples (**Figure 5D**). The correlation scatter plot was generated to visualize the associations between hub genes and immune cell infiltration levels. As depicted in **Figure 5E**, for LUAD samples, TMEM132A exhibited a strong positive correlation with resting mast cells, neutrophils, resting CD4 memory T cells, macrophages M2, monocytes, and resting NK cells, and a pronounced negative correlation with macrophages M1, plasma cells, and T follicular helper cells. Otherwise, TIMP1 showed a significant positive correlation with monocytes, neutrophils, and resting NK cells, while being negatively correlated with naive B cells, macrophages M1, activated CD4 memory T cells, and T follicular helper cells. In VTE samples, as shown in **Figure 5F**, TMEM132A was positively associated with naive B cells and activated CD4 memory T cells, and negatively with macrophages M0. TIMP1 exhibited positive correlations with naive B cells and resting CD4 memory T cells, and negative associations with macrophages M0, monocytes, and regulatory T cells. These results suggest that the immune infiltration environment of LUAD and VTE is different, and also emphasize the profound impact of TMEM132A and TIMP1 expression on the immune status of LUAD and VTE.

### Validation of hub gene

**Figure 6:**
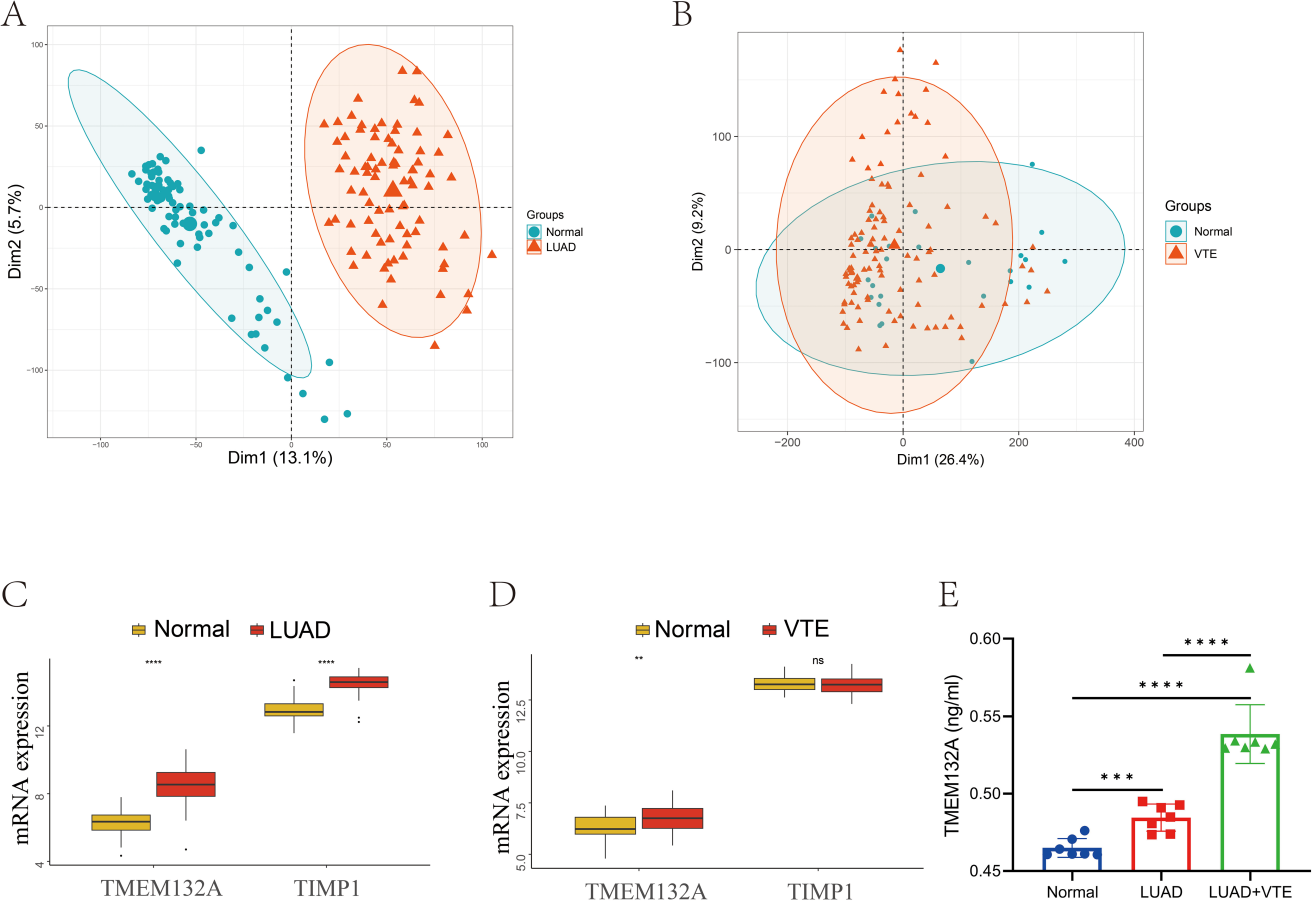
Validation of hub genes in GEO database and ELISA. **(A)** Sample distribution characteristics of PCA results of GSE75037. **(B)** Sample distribution characteristics of PCA results of GSE48000. **(C)** The expression level of hub genes in GSE75037. **(D)** The expression level of hub genes in GSE48000. **(E)** Verification of serum TMEM132A expression levels in human samples. **p < 0.01; ***p < 0.005; ****p < 0.001; ns, No statistical.

In order to further verify hub genes, the expression levels of the two hub genes were verified in the LUAD validation dataset GSE75037 and the VTE validation dataset GSE48000 respectively. Firstly, we performed PCA principal component analysis on the LUAD and VTE validation datasets. The results showed that the samples in the disease group and control group were clearly distinguished (**Figures 6A, B**). In the validation datasets, both TMEM132A and TIMP1 demonstrated significant upregulation in LUAD, as depicted in **Figure 6C**. In VTE samples, while TMEM132A showed significant upregulation, TIMP1 displayed downregulation without statistical significance (**Figure 6D**).

In light of the validation dataset outcomes, an ELISA was employed to examine TMEM132A serum levels in 7 patients with advanced LUAD, 7 with advanced LUAD combined with VTE, and 7 healthy subjects, to confirm whether TMEM132A is a core hub gene for LUAD-associated VTE. The results depicted in **Figure 6E** demonstrated that TMEM132A levels were significantly elevated in the LUAD group compared to the healthy controls (*p* < 0.001). Similarly, TMEM132A levels were markedly higher in the LUAD combined with VTE group compared to healthy controls (*p* < 0.0001). Notably, there was also a significant difference between the LUAD and LUAD combined with VTE groups (*p* < 0.0001).

### Evaluation of the diagnostic value of TMEM132A

**Figure 7:**
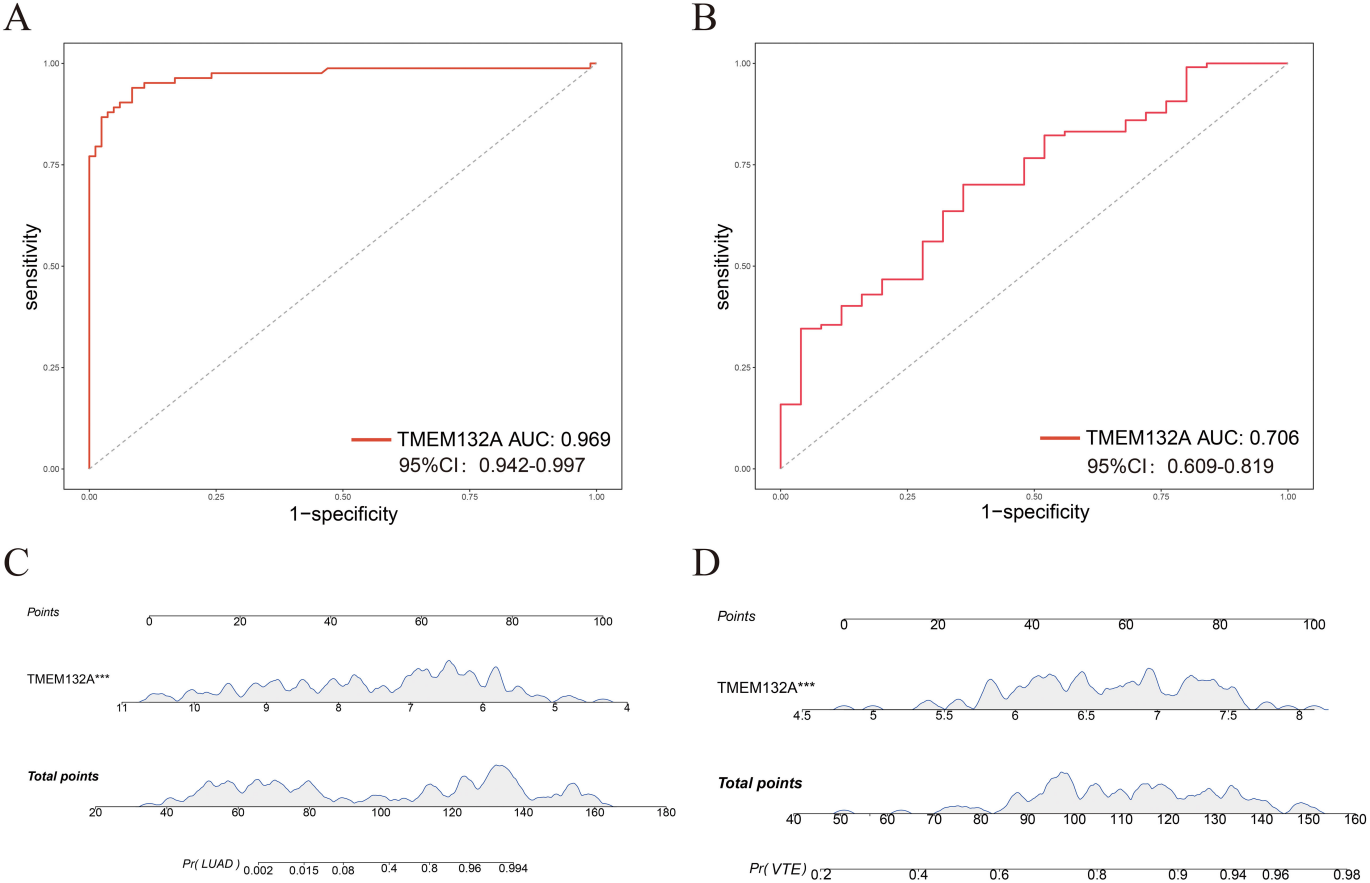
Evaluation of the diagnostic value of hub genes. **(A)** ROC curve analysis of TMEM132A in GSE75037. **(B)** ROC curve analysis of TMEM132A in GSE48000. **(C)** Nomogram predicting the probability of LUAD. **(D)** Nomogram predicting the probability of VTE. ***p < 0.005.

ROC curves were constructed to verify the diagnostic efficacy of TMEM132A in LUAD dataset and VTE dataset. In the LUAD dataset GSE75037, the AUC of TMEM132A was 0.969 (**Figure 7A**). In the VTE dataset GSE48000, the AUC of TMEM132A was 0.706 (**Figure 7B**). Nomogram was also constructed for TMEM132A, as illustrated in **Figures 7C, D**. This indicates that TMEM132A has strong diagnostic performance in LUAD and VTE. The above results also indicate that TMEM132A may be a biomarker for LUAD combined with VTE.

### Identification of candidate drugs based on the core hub gene

Using the DSigDB library in Enrichr, six drugs with significant *p*-value were screened out by calculating *p*-value and combined score to TMEM132A, indicating that these potential small molecule compounds may be used as co-treatments for LUAD and VTE (**Table 2**).

**Table 2:** Identification of candidate drugs for the treatment of LUAD and VTE based on the hub genes.

Drug	*P*-value	Odds Ratio	Combined Score
Birabresib	9.49E-06	10.54777702	121.982554
Abemaciclib	9.92E-06	10.47296683	120.6560008
MLN 8054	0.007438995	16.90136054	82.83389842
Vandetanib	0.002190906	7.85515873	48.10059473
Vorinostat	0.016262406	10.95587424	45.12614153
Dasatinib	0.003801442	6.699525745	37.33226907

## Discussion

VTE, encompassing DVT and PE, represents a prevalent and lethal pathophysiological state characterized by a significant mortality rate and a substantial incidence of misdiagnosis (29). A prospective study showed that patients with lung cancer had a high incidence of cancer-related thrombosis within 1 year (HR: 6.9, 95% CI: 3.0–15.9) (30). Pastori D et al. showed that the incidence of VTE in non-small cell lung cancer is 74.4 cases per 1,000 person-years (31). In a meta-analysis, LUAD was confirmed to be a risk factor for thrombosis in patients with lung cancer (OR: 2.20, 95% CI: 1.68–2.88) (32). Here, we utilize the existing public databases conducting this study to investigate the common characteristics and molecular pathways of potential CGs between LUAD and VTE from a bioinformatics perspective. This approach aims to offer a novel insight into the pathogenesis of these two diseases.

Here, 381 CGs of LUAD and VTE were identified. GO and KEGG enrichment analysis results showed that CGs is mainly involved in inflammatory response, immune regulation, and coagulation pathways. Ding Z et al. demonstrated that inflammation plays a key role in the development of LUAD (33). Inflammation creates a tumor-promoting microenvironment by inducing angiogenesis, suppressing adaptive immunity, and promoting tumor cell survival and proliferation (34, 35). In addition, inflammation also plays an important role in the occurrence and development of VTE (36). Under inflammatory conditions, the contact system is activated by activating FXII, which also results in the loss of some negative regulatory mechanisms (36). In the context of coagulation, FXIIa plays a dual role by not only activating the extrinsic pathway of FXI to generate thrombin but also by initiating the prekallikrein-kallikrein pathway, resulting in the production of plasma kallikrein (PK). Subsequently, PK triggers multiple potent pro-inflammatory pathways (37, 38). Inflammatory processes elevate levels of plasminogen activator inhibitors, leading to reduced plasminogen activity, disturbances in fibrinolysis, and enhanced thrombus formation (37, 39). Therefore, inflammation may play a very critical role in the occurrence of VTE in LUAD patients, this is consistent with our conclusion.

LASSO is a machine learning technique that combines variable selection and regularization to improve prediction accuracy (40). This study finally obtained two hub genes (TIMP1 and TMEM132A) through lasso regression. Subsequently, a nomogram was developed, and ROC analysis was conducted to assess the diagnostic capabilities of the hub genes. Analysis of immune cell infiltration revealed significant associations with these hub genes. Expression levels of these genes were confirmed in a validation dataset. Furthermore, the core hub gene underwent additional validation through clinical sample testing. The findings ultimately revealed that, within the validation cohort, TIMP1 exhibited no significant differences in the context of VTE, whereas substantial disparities in TMEM132A expression were observed between the LUAD and VTE validation datasets. Studies have shown that TIMP1 can be used as a soluble marker for pancreatic cancer (41). In addition, TIMP1 expression can also predict new postoperative atrial fibrillation (42). In thrombosis, TIMP1 has been shown to be significantly associated with DVT (43). The results of the study on TIMP1 were inconsistent with those of this study, but TMEM132A was shown to be associated with poor prognosis of tumors and was significantly correlated with the infiltration of multiple immune cells (44, 45). This evidence strongly suggests that TMEM132A is a key gene common to both LUAD and VTE.

TMEM132A is a multifunctional transmembrane protein which plays a key role in many physiological and pathological processes such as cell adhesion, signal transduction, cancer development and neurodevelopment. The protein has immunoglobulin-like domains and leucine-rich repeat domains, which provide the structural basis for its functions in cell adhesion and signal transduction (46, 47). Notably, TMEM132A is known to interact with Wntless (WLS), a pivotal protein in the Wnt signaling pathway. This interaction impacts the transport and secretion of Wnt ligands, which are vital for the regulation of the Wnt signaling pathway (48, 49). Furthermore, knockdown of TMEM132A inhibits Wnt/β-catenin signaling, resulting in a phenotype similar to that of Wnt/β-catenin mutations (50). The Wnt/β-catenin signaling pathway plays a crucial role in regulating cell proliferation, differentiation, apoptosis, tissue homeostasis, and wound healing. Various cancers are intimately linked to the dysregulation of this factor (51). In LUAD, TMEM132A expression was markedly elevated, showing a strong positive correlation with processes such as differentiation and angiogenesis, while exhibiting a negative correlation with DNA repair and cell cycle activities (44). Our findings are consistent with established data, demonstrating that the serum levels of TMEM132A were significantly elevated in both the LUAD cohort with and without VTE, compared to those in the healthy control group.

The pathophysiological mechanisms of VTE in lung cancer patients are encapsulated by Virchow’s triad, which highlights three critical factors: blood stasis, hypercoagulability, and endothelial dysfunction or injury. The interplay of these elements in the context of lung cancer continues to fuel debates regarding the precise mechanisms underlying thrombosis (52). Research indicates that patients with lung cancer often exist in a hypercoagulable state. The occurrence of this condition is partly attributed to the tumor itself, as well as to the activation of endothelial and inflammatory cells, which subsequently release substantial quantities of procoagulant substances, such as tissue factor. These substances directly initiate the coagulation cascade, significantly increasing the risk of thrombosis (53, 54). Research shows unregulated reciprocal activation of the immune and coagulation systems can lead to catastrophic thrombotic and inflammatory damage (36). Monocytes interact with the endothelium in host defense and play a key role in the development of VTE (55). The results of this study are consistent with the observation that monocytes and macrophages M0 were significantly increased in VTE samples. Furthermore, TMEM132A exhibited a significant positive correlation with naive B cells and activated CD4^+^ memory T cells in these VTE samples. The TMEM132A protein may influence the immune response in LUAD by modulating immunoregulatory genes and affecting the infiltration of immune cells (44). In this investigation, we observed a significant positive association of TMEM132A with resting mast cells, neutrophils, resting CD4^+^ memory T cells, M2 macrophages, monocytes, and resting natural killer (NK) cells within LUAD samples. Studies have shown that activation of the Wnt/β-catenin signaling pathway can promote the polarization of M2 macrophages (56). However, inhibition of the Wnt/β-catenin signaling pathway can increase the infiltration of CD4^+^ T cells and CD8^+^ T cells (56, 57). In addition, studies have found that activation of the Wnt/β-catenin signaling pathway can also cause infiltration of resting CD4^+^ T memory cells (58). Also, high expression of TMEM132A can activate the Wnt/β-catenin signaling pathway, and the regulatory role of the Wnt/β-catenin signaling pathway in various diseases, including the tumor microenvironment, has been proven (49, 50). This means that the effect of TMEM132A on immune cell infiltration may be achieved through the activation of the Wnt/β-catenin signaling pathway, but this potential mechanism needs further proof. Consequently, TMEM132A may contribute to immune regulation, and its role in immune responses could be crucial in the occurrence of VTE among LUAD patients via Wnt/β-catenin signaling pathway. Additionally, by influencing the Wnt signaling pathway, TMEM132A facilitates cell adhesion and proliferation, suggesting its potential involvement in the pathogenesis of VTE in LUAD patients.

In this research, the data revealed that TMEM132A serum levels in the LUAD group combined with VTE were markedly elevated compared to those in the LUAD-only group and healthy individuals. This suggests that TMEM132A could represent a significant risk factor for VTE occurrence in LUAD patients. Moving forward, we aim to explore the underlying mechanisms by which TMEM132A may facilitate VTE onset in LUAD patients.

Nonetheless, this investigation has certain limitations. While TMEM132A was validated via ELISA, the quantity of clinical samples employed was restricted. Future *in vivo* experiments and expanded clinical sample sizes will be necessary to confirm our findings.

## Conclusion

This investigation identified two hub genes (TIMP1 and TMEM132A), of which only TMEM132A demonstrated substantial diagnostic efficacy in LUAD and VTE. This confirms TMEM132A as a key gene common to both conditions, potentially linked to an increased likelihood of VTE occurrence in LUAD patients. Analysis of immune infiltration revealed a significant association between this key gene and immune cells, indicating that TMEM132A may influence the immune microenvironment in LUAD and VTE. The findings offer novel perspectives on the shared mechanisms and interrelations between LUAD and VTE.

## Data Availability

Publicly available datasets were analyzed in this study. The datasets used in this study were from the GEO database (GSE10072, GSE32863, GSE40791, GSE43458, GSE46539, GSE75037 and GSE48000). VTE-related genes were sourced from four databases: the CTD (http://ctdbase.org/), DisGeNET (https://www.disgenet.org/), GeneCards (https://www.genecards.org/), and OMIM (http://omim.org/).
